# Overcoming treatment resistance in cholangiocarcinoma: current strategies, challenges, and prospects

**DOI:** 10.3389/fcell.2024.1408852

**Published:** 2024-08-02

**Authors:** Jiayi Wang, Siyan Liu, Yi Cao, Yong Chen

**Affiliations:** ^1^ International Medical College, Chongqing Medical University, Chongqing, China; ^2^ Second Clinical College, Chongqing Medical University, Chongqing, China; ^3^ Department of Hepatobiliary Surgery, The First Affiliated Hospital of Chongqing Medical University, Chongqing, China

**Keywords:** resistance in cholangiocarcinoma, targeted therapies, treatment of cholangiocarcinoma, single-cell and spatial transcriptomic perspective, cholangiocarcinoma (CCA)

## Abstract

Significant advancements in our understanding and clinical treatment of cholangiocarcinoma (CCA) have been achieved over the past 5 years. Groundbreaking studies have illuminated the immune landscape and pathological characteristics of the tumor microenvironment in CCA. The development of immune- and metabolism-based classification systems has enabled a nuanced exploration of the tumor microenvironment and the origins of CCA, facilitating a detailed understanding of tumor progression modulation. Despite these insights, targeted therapies have not yet yielded satisfactory clinical results, highlighting the urgent need for innovative therapeutic strategies. This review delineates the complexity and heterogeneity of CCA, examines the current landscape of therapeutic strategies and clinical trials, and delves into the resistance mechanisms underlying targeted therapies. Finally, from a single-cell and spatial transcriptomic perspective, we address the challenge of therapy resistance, discussing emerging mechanisms and potential strategies to overcome this barrier and enhance treatment efficacy.

## Introduction

Cholangiocarcinoma (CCA) represents a highly lethal epithelial carcinoma within the hepatobiliary system, usually classified as intrahepatic, perihilar, and distal based on various anatomical locations (Brindley et al., 2021). CCA is the second most common liver cancer accounting for 15%–20% of all primary liver cancers (Gingold et al., 2018). In contrast to hepatocellular carcinoma (Xue et al.), the rare incidence of it has in fact increased the complexity and challenge of treatment (Balogh et al., 2016). The late diagnoses and poor prognoses are an obstacle to further improvement of therapeutic effectiveness: patients have an overall 5-year overall survival (Bridgewater et al.) ranges from 7% to 20% (Balogh et al., 2016; Banales et al., 2019; Zhu and Kwong, 2020).

Considering the continuing high recurrence and rapid progression after treatment worldwide, understanding the risk factors for CCA is essential to improve therapy efficacy. In Thailand with the highest incidence (30–40 out of 100,000), the top one pathogenic factor is the infection of liver fluke (Bridgewater et al., 2014). In contrast, in western countries with relatively low incidences (Xue et al., 2019), risk factors are diverse and usually include hepatitis B/C virus, fatty liver, alcohol, and biliary inflammation (Palmer and Patel, 2012; Bridgewater et al., 2014). Recently, a few studies pointed out wider risk factors for CCA in a perspective of liver diseases such as fibroinflammatory biliary duct diseases and primary sclerosing hepatitis (Razumilava and Gores, 2014; Bertuccio et al., 2019; Kelley et al., 2020).

In our recent summary and review, we have consolidated significant advancements, including studies utilizing next-generation sequencing, single-cell sequencing, spatial transcriptomic sequencing, and other multi-omics analyses. These studies have provided insights into the mechanisms of CCA resistance, addressing aspects such as the identification of driver genes, challenges related to specific target resistance, cell-cell interactions within the tumor microenvironment, and the spatial heterogeneity of tumors. This review underscores the critical issue of therapeutic resistance and the development of novel combination treatment strategies. The objective is to innovate therapeutic approaches and improve the adverse clinical outcomes associated with CCA.

### Advances in the genomic landscape and laboratory technology of CCA

**FIGURE 1:**
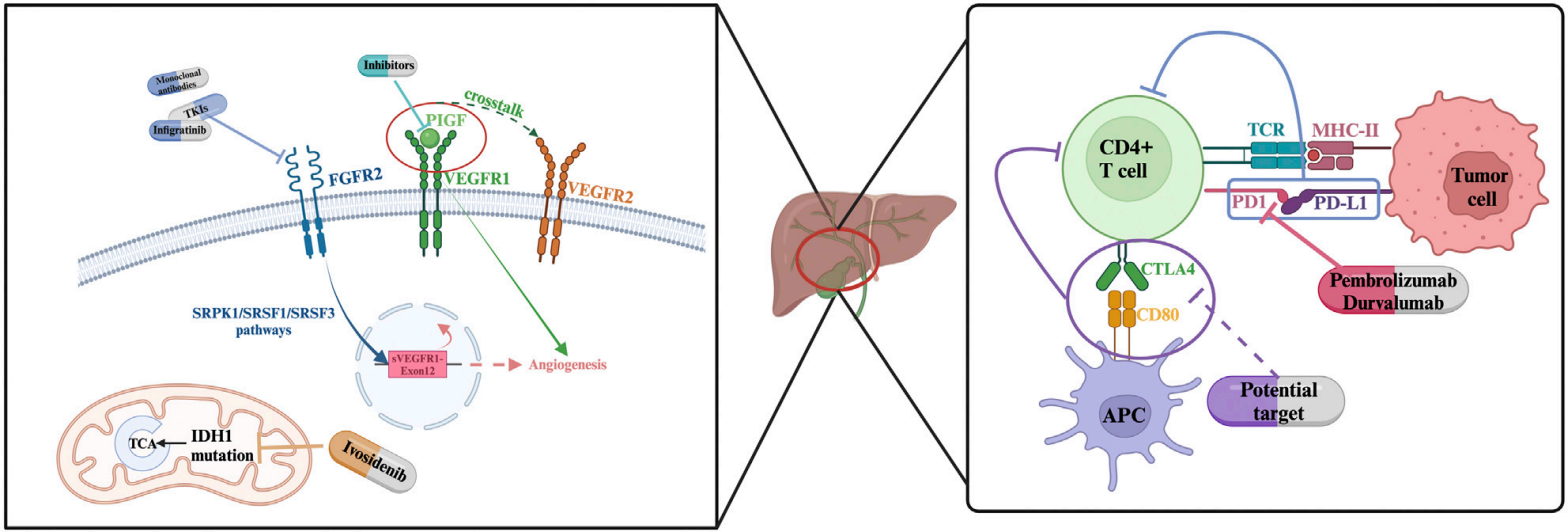
General condition of targeted therapy for cholangiocarcinoma.

Our understanding of the genomic landscape of cholangiocarcinoma (CCA) has significantly deepened (Figure 1). Since 2013, extensive next-generation sequencing efforts have identified diverse subgroups of intrahepatic cholangiocarcinoma (ICC) for clinical consideration. Notably, Sia, Moeini, and Montal, along with their teams, have made significant contributions to elucidating the molecular signatures and actionable targets in CCA at various localizations. In 2013, Sia et al. identified two types of ICC—proliferation and inflammation—by analyzing signaling pathways activated in tumors of specific molecular classes and copy number variation (Sia et al., 2013).

In 2014, Gao and colleagues reported a whole-exome sequencing (Schneider et al.) study linking PTPN3 mutations to CCA proliferation, migration, and recurrence potential (Gao et al., 2014). The Lawrence Kwong lab at MD Anderson Cancer Center contributed to The Cancer Genome Atlas (Farshidfar et al.), performing comprehensive analyses of somatic mutations, DNA methylation, whole-genome expression, and copy number variation. Their work highlighted the isocitrate dehydrogenase (IDH) mutation as a stratification marker for the CCA population (Farshidfar et al., 2017). Kwong’s work is regarded as a cornerstone in ICC bulk sequencing studies.

In 2019, researchers from China, Japan, and Singapore conducted a joint study sequencing the genome and transcriptome of 133 East Asian patients, elucidating CCA heterogeneity and providing molecular subtypes for pathological reference (Xue et al., 2019). In 2022, Gao, Q.’s lab performed in-depth sequencing on different spots for each patient, delineating the heterogeneity of immune infiltration in CCA (Lin et al., 2022). Additionally, Sia’s team provided comprehensive molecular characterization and identified multiple subtypes of mixed hepatocellular-cholangiocarcinoma (HCC-ICC) and extrahepatic cholangiocarcinoma (eCCA) in 2017 and 2020, respectively (Moeini et al., 2017; Montal et al., 2020). These studies have facilitated an in-depth exploration of the molecular mechanisms of CCA across different locations and types.

Advancements in laboratory technology have also been noteworthy. The study by Calvisi DF and Chen X in 2014 achieved stable reproduction of mouse liver cancer models through hydrodynamic tail vein injection (HTVI) technology. They demonstrated that liver tumors could be induced by transfecting oncogenes and tumor suppressor genes into hepatocytes, replicating specific pathological environments and tumor progression periods (Chen and Calvisi, 2014). This method allows for the efficient delivery of oncogenes, eliminating the need for breeding transgenic mice to study liver tumors. Consequently, new insights into the pathology and origin of hepatocellular carcinoma and intrahepatic CCA have been revealed.

### Standard treatment

Understanding the current clinical treatment landscape for cholangiocarcinoma is crucial for recognizing the significance of immunotherapy. Standard treatment strategies for CCA include surgical resection, systemic chemotherapy, and combinations of chemotherapy with targeted therapy. For intrahepatic cholangiocarcinoma (iCCA), only 30%–40% of cases are suitable for surgical resection. Even among those who undergo early surgical resection, the recurrence rate remains higher than 50% (Bridgewater et al., 2014). Monotherapy chemotherapy regimens have also shown limited success. Since 2019, two phase III randomized studies assessing adjuvant therapy with gemcitabine alone after surgical resection for CCA and gallbladder cancer (GBC) reported no significant improvement in survival rates (Primrose et al., 2019; Lamarca et al., 2020b; Lamarca et al., 2022).

In the quest to improve survival rates and immune response in CCA, resistance to targeted therapy inhibitors remains a significant challenge. This review aims to elucidate the mechanisms behind inhibitor resistance and the pathways leading to immune escape in CCA, with the goal of identifying new targets and perspectives for future treatments. A substantial barrier to the development of targeted therapies for CCA is the general lack of a predominant oncogenic driver in many cases, limiting the subset of patients who could benefit from these therapies. However, targeted treatment can still be relevant for patients harboring specific mutations, with potential targets including Isocitrate Dehydrogenase 1/2 (IDH1/2), Fibroblast Growth Factor Receptor 2 (FGFR2), Neurotrophic Receptor Tyrosine Kinase (NTRK), HER2, BRAF, ROS, and RET (Lamarca et al., 2020a; Harding et al., 2023).

Enhanced by the availability of open-source bulk sequencing and single-cell RNA sequencing data, combination therapies that include targeted treatments are showing promise. In the realm of first-line treatments, there is growing evidence that combining the immune checkpoint inhibitor durvalumab, a Programmed Death-Ligand 1 (PD-L1) inhibitor, with gemcitabine and cisplatin can significantly improve survival rates, establishing a new standard of care for patients with advanced-stage CCA (O'Rourke et al.). In the subsequent sections, we explore a range of prominent therapeutic agents, from inhibitors developed for traditional targetable molecules to immune checkpoint inhibitors (ICIs). This discussion encompasses drugs currently in clinical trial phases as well as those that have received regulatory approval. We delve into the phenomena of resistance to inhibitors and ICIs, highlighting several studies investigating the underlying mechanisms of resistance. Furthermore, we propose potential pathways to overcome this resistance, offering insights into future strategies for enhancing the efficacy of CCA treatments.

### Molecular targeted therapy

In recent years, clinical trials targeting specific molecules have provided good support and information for the introduction of new treatment options into clinical practice. Table 1 summarizes some of the clinical trials that have recently ended and are ongoing. In the following sections, we further discuss some of these and other CCA clinical results that have attracted attention in recent years.

**TABLE 1:** Cholangiocarcinoma clinical trials.

Trial code	Study arm	Study phase	Inhibitor	Primary endPoint(s)	ORR (advances)
TOPAZ-1 (Oh et al., 2022)	durvalumab	Phase III	PD-L1	OS	26.7%
FIGHT-202 (Abou-Alfa et al., 2020b)	Pemigatinib	Phase II	FGFR2	ORR	35.5%
FIGHT-302 (Bekaii-Saab et al., 2020)	Pemigatinib	Phase III	FGFR2	OS, ORR	-ongoing
BGJ398 (Javle et al., 2021a)	Infigratinib	Phase II	FGFR2	ORR	23.1%
ClarIDHy (Abou-Alfa et al., 2020a)	Ivosidenib	Phase III	IDH1	PFS	2–7 months
KEYNOTE-158 (Marabelle et al., 2020)	Pembrolizumab	Phase II	MSI-H/dMMR	ORR	34.3%
LEAP-005 (Perez-Fidalgo and Martinelli, 2023)	Lenvatinib + pembrolizumab	Phase II	ICIs	ORR	22%
FIDES-01 (Park et al., 2019)	Derazantinib	Phase II	FGFR2	ORR	-ongoing
FOENIX-CCA2 (Goyal et al., 2021b)	Futibatinib	Phase II	FGFR2	ORR	41.7%
ECOG-ACRIN EA6134 (Atkins et al., 2023)	Dabrafenib + trametinib	Phase III	BRAF	ORR, OS	47.8%–29.6%

### FGFR2 Inhibitors: clinical trial, toxicity, resistance mechanisms, and revisit of clinicogenomic analysis

Fibroblast growth factor receptor (FGFR) encompasses a family of tyrosine kinase receptors instrumental in the regulation of cellular proliferation and growth (Turner and Grose, 2010). Genetic alterations such as fusions and rearrangements of FGFR2 occur in 10%–15% of intrahepatic cholangiocarcinoma (iCCA) cases but are rarely observed in extrahepatic cholangiocarcinoma (Cadamuro et al., 2019; Goyal et al., 2021a). Pemigatinib, the first therapy approved by the U.S. FDA for the treatment of advanced CCA patients with FGFR2 fusion and rearrangement positivity, demonstrated objective responses in 38 out of 146 enrolled patients (Liu et al., 2020). Furthermore, other targeted drugs developed for the FGFR pathway are in clinical trial phases, including selective and non-selective FGFR2 tyrosine kinase inhibitors (TKIs), and anti-FGF/FGFR monoclonal antibodies (Ghedini et al., 2018). Numerous studies reporting on the clinical efficacy of targeting FGFR2 fusion positive CCA have been published. Promising agents such as Debio 1,347, Derazantinib, Erdafitinib, and Infigratinib are currently undergoing phase II-III studies, with some of the results reported as of the writing of this article (Park et al., 2019; Cleary et al., 2020; Goyal et al., 2021a; Javle et al., 2021b). For patients with refractory advanced CCA harboring FGFR2 fusions or rearrangements, these therapies have demonstrated objective response rate (ORR) ranging from 20.7% to 47%. A multi-national, single-group, phase II study published in 2023 indicated that the covalent FGFR inhibitor futibatinib provided objective clinical benefits to patients with iCCA who had FGFR2 fusions or rearrangements and who had previously undergone treatment (Goyal et al., 2023).

Despite FDA has approved multiple FGFR2 inhibitors in clinical treatment, unsatisfied ORRs (<45%) were seen upon this type of treatment so far. Progression-free periods are maintained at 6–12 months and there is evidence that this may be associated with acquired alterations in the FGFR2 kinase domain and FGFR inhibitors disfunction of binding (Byron et al., 2013; Goyal et al., 2017; Goyal et al., 2019; Krook et al., 2019; Silverman et al., 2021; Varghese et al., 2021). FGFR1-3 inhibitor, Futibatinib (TAS-120) remains efficacy against a series of secondary FGFR2 mutations, though it is not effective against all (Goyal et al., 2019). Besides, further evidence indicates that FGFR inhibitor resistance could be still gained under circumstances of no occurrence of genetic alterations or those caused by other MARK signaling components (Goyal et al., 2021b; Cleary et al., 2021). Pharmacodynamically, almost all FGFR inhibitors are associated with increased phosphate levels; hence, concomitant phosphate-lowering treatment may be necessary to patients experiencing hyperphosphatemia during FGFR inhibitor therapy. Additionally, various degrees of ocular and nail abnormalities have been reported in these studies as side effects of the treatment (Javle et al., 2018; Abou-Alfa et al., 2020b; Goyal et al., 2020; Xie et al., 2020). All the enigma underscores the importance of further investigating FGFR inhibitors resistance mechanisms.

Efforts to explore resistance mechanisms to FGFR inhibitors and ways to overcome this resistance have been proposed and are under exploration. It is currently understood that there are two distinct FGFR2 resistance acquisition types: primary resistance and acquired resistance. Silverman and colleagues reported observations that individuals with FGFR2 fusion, who also possess tumor suppressor gene alterations (including BAP1, CDKN2A/B, PBRM1, and TP53), have shorter progression-free survival (Silverman et al., 2021). Regarding acquired resistance, one study reported the emergence of an FGFR2 V565F gatekeeper mutation in patients with FGFR2 fusion iCCA treated with infigratinib. Furthermore, two patients were reported to have developed multiple polyclonal secondary mutations (Goyal et al., 2017).

To combat resistance, a batch of influential research was conducted in aspects of increasing FGFR inhibitor sensitivity, focusing on potential efficient inhibitors, and presenting molecular landscape in recent few years. Wu and colleagues performed high-throughput combination drug screens (Wu et al., 2022b) on patient-derived xenograft (PDX) cell lines and mouse models. Their results illustrate that adaptive signaling through EGFR plays a key role in lowering FGFR inhibitor sensitivity and developing resistance. In sensitive models, disturbed cell death induction processes are observed. While suppressing wild-type EGFR responses through inhibiting MEK/ERK and mTOR signaling causes cell death and tumor regression. Another influential study was published on Sept. 06, 2023, and it recorded the team’s discovery of the highly selective, irreversible, small-molecule RLY-4008s capability of inducing tumor regression and focusing both primary and acquired resistance (Subbiah et al., 2023).

Beyond the exploration of potential mechanisms of resistance, Silverman et al. conducted sequencing on a clinical cohort of cholangiocarcinoma (CCA) with FGFR2 rearrangements, providing direct evidence of the response to Pemigatinib targeted therapy. In this work, a post-treatment clinical genomic landscape was constructed for the FIGHT-202 cohort, examining the response of patients with FGFR2 fusions or rearrangements and those without FGFR2 alterations to Pemigatinib, as well as investigating pathways to acquired resistance to Pemigatinib. These findings offer invaluable insights for the application of Pemigatinib and future FGFR2-targeted therapies via suggesting a wide range of selection and enlightenment of potentiality of FGFR2 inhibitors’ resistance acquisition (Silverman et al., 2021).

### IDH1/IDH2 inhibitors: clinical insights, and resistance mechanisms

Isocitrate dehydrogenase (IDH) plays a pivotal role in cellular metabolic processes. Alterations in the genes responsible for the metabolic enzymes IDH1 and IDH2 have been linked to the emergence of early-stage biliary lesions with potential malignancy (Valle et al., 2017; Hadfield et al., 2023). Mutations in IDH1/2 are found in approximately 13%–36% of intrahepatic cholangiocarcinoma cases and are less common in extrahepatic cholangiocarcinoma, constituting less than 1% of instances (Valle et al., 2017; Boscoe et al., 2019; Abou-Alfa et al., 2020a). Mutations in Isocitrate Dehydrogenase 1/2 (IDH1/2) are frequently observed in cholangiocarcinoma (CCA). Ivosidenib (AG120), a small molecule and selective inhibitor, has been developed targeting the IDH1 mutation and has recently been approved by the FDA for use in advanced-stage and metastatic intrahepatic cholangiocarcinoma (iCCA) (Norsworthy et al., 2019; Zhu and Kwong, 2022). However, phase III trial reports indicate that the objective response rate (ORR) and disease stability (SD) are only 2% and 51% respectively (Zhu et al., 2021). Moreover, IDH1 mutation-driven mouse models of similar solid tumors do not exhibit tumor regression upon removal of the IDH1 mutation, implying a limited efficacy of IDH1 mutation inhibitors when used as monotherapy in tumors with comparable pathological conditions (Turcan et al., 2018).

In response to the challenges of low sensitivity and resistance faced by IDH mutation inhibitors, Wu and colleagues have engineered mouse models with IDH1 mutations and uncovered that tumor maintenance is mediated by dual (R)-2-hydroxyglutarate activities: suppression of CD8^+^ T cell activity and the autonomous inactivation of TET2 DNA demethylase within tumor cells (Wu et al., 2022a). This epigenetic and metabolic shift in the tumor microenvironment, as demonstrated in these mouse models, suggests that immune checkpoint blockade coupled with the IFN-γ-TET2 axis can surmount immunosuppression, thereby providing a strategy to counteract the resistance to IDH1 mutation inhibitors. Kwong et al. have investigated the potential synergistic effects of combining PD-L1 inhibitors with AG120 (ivosidenib) and the pairing of CTLA4 antibodies with AG120 in their research (Zhu and Kwong, 2022). Their findings reveal that while the combination of PD-L1 inhibitors and AG120 does not result in a synergistic effect, such an effect is evident between CTLA4 antibodies and AG120. Building upon previously reported studies, their exploration proposes a new avenue: the combination of CTLA4 inhibitors and IDH1 mutation-targeted treatments as a promising therapeutic strategy.

### VEGF inhibitors and PIGF

Vascular endothelial growth factor (VEGF) modulates cancer cell proliferation with its functional role in enhancing angiogenesis. VEGF has been shown to be upregulated in over 75% hepatobiliary malignancies (Valle et al., 2017). VEGF inhibitor sorafenib combinedly used with gemcitabine are proven to provide clinical benefits to unresectable or metastatic BTC patients (Moehler et al., 2014). Several studies have shown that high expression of VEGF receptor (VEGFR) in a hypoxic environment can induce tumor angiogenesis, and Hypoxia inducible factor 1 alpha (HIF-1a) induces the production of multiple mediators in hypoxia. Therefore, inhibitors against VEGF have become an important issue. (Ramakrishnan, 2014 #145).

Placental growth factor (PlGF), a constituent of the vascular endothelial growth factor (VEGF) family, typically engages with Nrp1 and VEGFR1 on the surface of endothelial cells. This interaction promotes crosstalk among Nrp1, VEGFR1, and VEGFR2, thereby amplifying the cellular responses initiated by VEGF (Simons et al., 2016; Aoki et al., 2022). PIGF has been identified as a signal molecule that activates tumor cells, prompting the recruitment of stromal cells and subsequently enhancing angiogenesis and inflammatory responses. PlGF is closely associated with the tumor progression and metastasis (Fischer et al., 2007; Rolny et al., 2011; Heindryckx et al., 2013). As a potential marker for inhibiting the proliferation of cholangiocarcinoma cells, PlGF presents favorable conditions due to its high expression under pathological and hypoxic conditions, which allows for the selective inhibition of pathological angiogenesis (Green et al., 2001; Jain and Xu, 2007).

Zhu and colleagues reported Nrp1’s critical role in restricting CCA tumor cell proliferation and migration, and further inhibiting the tumor progression and lung metastasis *in vitro* and vivo experiments (Zhu et al., 2018). A novel study explored the potential mechanisms of PIGF overcoming chemotherapy insensitivity (Aoki et al., 2022). This study demonstrates the potential of Placental Growth Factor (PlGF) blockade in ameliorating the hypoxic conditions within solid tumors, improving blood perfusion, and enhancing the sensitivity to standardized chemotherapy regimens.

Moreover, inhibitors developed for rare mutation-driven targets with low occurrence in the population have also shown objective improvements. Neurotrophic tyrosine receptor kinase (*NTRK*) fusion inhibitors entrectinib and Larotrectinib are approved for advanced-stage solid tumor patients in 2019 (US Food and Drug Administration, 2018; US Food and Drug Administration, 2019). In 2022, the combination of dabrafenib and trametinib (targeting BRAFV600E mutation) is approved for unresectable or metastatic CCA (US Food and Drug Administration, 2022). Molecular therapies targeting various mutation points have been extensively explored through clinical trials and evaluations of therapeutic effects. The next step is to review the current state of Immune Checkpoint Inhibitor (O'Rourke et al.) treatments in recent years, summarize the significant work on studying ICI resistance mechanisms from a single-cell perspective, enhance our understanding of how to overcome resistance, and identify potential paths to improve the response rate.

### ICI rechallenge and strategies: clues from the single cell perspective

#### ICIs current status and efficacy

Immune checkpoint inhibitors (ICIs) primarily refer to agents such as cytotoxic T-Lymphocyte antigen-4 (CTLA-4) and programmed death-1/programmed death-ligand-1 (PD-1/PDL-1), which are antibodies that block immune checkpoint proteins (Hadfield et al., 2023). Immune checkpoint inhibitors therapy represents a novel therapeutic approach, particularly for malignant tumors. Patients with high microsatellite instability (MSI-H) or deficient DNA mismatch repair (dMMR) are especially targeted for this type of treatment. In 2017, the United States Food and Drug Administration (FDA) first approved the PD-1 inhibitor pembrolizumab for the treatment of these two types of tumors. (Wang et al., 2021). In gastrointestinal malignancy realm only, the incidence rates of MSI-H is ranging under 60% among all cases (Williams and Huang, 2013). In this phase II trial that included 22 patients with cholangiocarcinoma (CCA), the ORR for patients with MSI-H or dMMR was 40.9% (Marabelle et al., 2020). MSI-H are commonly found with incidence of chronic cholecystitis and pancreaticobiliary maljunction. Furthermore, pembrolizumab has received expanded approval for its therapeutic indications. In 2020, it was approved for the treatment of patients with high tumor mutational burden (TMB-H) solid tumors (US Food and Drug Administration, 2020). Dostarlimab is approved for the treatment of patients with dMMR who have recurrent or advanced-stage disease in adults. These patients receiving pembrolizumab treatment belong to the group with unresectable or metastatic solid tumors. In this context, pembrolizumab is used as a subsequent therapy to inhibit disease progression (US Food and Drug Administration, 2021).

Evaluation of the efficacy of ICIs in cholangiocarcinoma and other neighboring hepatobiliary carcinomas is ongoing. As previously mentioned, currently using chemotherapy combined with ICIs as a standard treatment strategy is a hot topic. A phase III study evaluated the combination of gemcitabine and cisplatin with the PD-1 inhibitor Durvalumab, which currently reports an ORR of 24.9%. In addition, in the phase II LEAP-005 study, advanced hepatobiliary carcinoma patients are shown to gain clinical benefits from the second-line treatment of the combination of pembrolizumab and Lenvatinib (Perez-Fidalgo and Martinelli, 2023). In the treatment of advanced hepatobiliary patients, the PD-1 inhibitor nivolumab has also demonstrated objective remission on dMMR patients, and therefore nivolumab can be used as a first-line treatment for future patients with such features.

### Diverse cells participate in tumor initiation and interact with the tumor microenvironment

The explosive prosperity of single-cell sequencing technologies has enabled us to observe the heterogeneity of the immune landscape and the metabolic microenvironment presented by cellular behaviors and pathological characteristics in cholangiocarcinoma (CCA) at a single-cell resolution. A series of influential single-cell RNA sequencing and spatial transcriptomics studies have become the key to our in-depth exploration of the resistance mechanisms to immune checkpoint inhibitors (ICIs). Next, we describe the insights into CCA on single cell perspective and give introduction to a batch of influential studies:

Ma and colleagues conducted single-cell transcriptomic sequencing on patients with hepatocellular carcinoma (HCC) and intrahepatic cholangiocarcinoma (ICC), studying the behavior of different cellular populations within the tumor microenvironment from the perspective of reprogramming in hepatobiliary cancers (Ma et al., 2019). This study identified an axis where the expression of vascular endothelial growth factor A (VEGFA) in malignant cells correlated with higher degrees of hypoxia; in conjunction with other studies, it suggests that the regulation of VEGFA induces levels of hypoxia-inducible factor 1-alpha (HIF1A) mRNA and protein, activating downstream hypoxic signaling pathways (Wiener et al., 1996; Semenza, 2012). The work discovered high expression of T cell toxicology-related genes (GZMA, GZMB, GZMH, and PRF1) and immune checkpoint genes PDCD1, IFNG, and NKG7 in CD8^+^ and CD4^+^ cells in the low diversity group (Div-low), indicating that tumors classified in this manner are potential effective targets for ICI therapy. Furthermore, the results imply that anti-angiogenic drug treatments may enhance the efficacy of immunotherapy (Khan and Kerbel, 2018); Another sequencing work targeting vascular cancer-associated fibroblasts (vCAFs) subgrouping reported tumor-infiltrating CD4 regulatory T cells performed high correlation with immune suppression (Zhang et al., 2020); Additionally, we are pleased to see direct comparisons of treatment failures and successes. This work has illustrated changes and distinctions in the tumor evolutionary trajectories post-treatment, providing new evidence for identifying possible causes of therapeutic failure (Ma et al., 2020). Tumor cell clonality is related to the polarization of CD4 T cells and CD8 T cells: memory and cytotoxic CD8 T cells are enriched in low clonality groups, while proliferative pre-exhausted and conventional pre-exhausted T cells are enriched in high clonality groups. CD8 T cells were enriched in downstream pathways associated with immune response. Through these studies we gained basic comprehension of common T cells’ functional role under single-cell vision, but it’s also important to notice other cell types’ function in CCA (Golino et al., 2023).

Accumulated evidence has revealed that not only cholangiocytes and hepatocytes but also other cells such as fibroblasts, endothelial, and tumor-associated macrophages (TAMs) participate in the pathological process and tumor initiation of cholangiocarcinoma in various ways (Sato et al., 2021). While the previous research on tumor initiation and development focused on the immune suppression and the unbalance of the tumor microenvironment, recent studies focus more on reporting how these cells in the iCCA microenvironment interact with the tumor structure and make alterations to the surrounding microenvironment (Zhang et al., 2020; Affo et al., 2021). These cells play a critical role in modulating the balance and pressure in the microenvironment.

In general, CCA tumor structure is a patchwork of fibrotic stroma, inflamed, gliotic tissue. A few types of cells enrich the microenvironment: cancer-associated fibroblasts (CAFs), T cells, B cells, endothelial, lymphatic cells, TAMs, Tregs, and NK cells (Fabris et al., 2019; Fabris et al., 2021) These cells promote CCA invasion and progress via inhibiting immune responses, inducting angiogenesis, or activating migration with certain signaling pathways. For instance, we will introduce a typical pair, CCA cells-CAFs, and the crosstalk in between. CCA cells release platelet-derived growth factor D (PDGF-D) causing CAFs to recruit in the close area of the tumor tissue. CAFs prompt further fibrosis of healthy cells and secrete vascular endothelial growth factors (VEGF) to induct lymphangiogenesis and angiogenesis in CCA (Cadamuro et al., 2019). CAFs’ pro-angiogenic effect under special circumstances enables CCA invasive and self-maintaining. Alternatively stated, the power that CCA fuels the maintenance and tumor development is from the tumor microenvironment itself after the formation.

Like the functions of CAF, TAMs play an analogous role in regulating CCA progression. In the tumor microenvironment, TAMs exist as the most enriched immune cells, and they play a pivotal role in modulating the tumor progression through participation in the crosstalk between malignant cells and the tumor microenvironment (Franklin and Li, 2016; Cortés et al., 2017). TAMs promote invasion in several aspects. First, TAMs of activated phase secrete cytokines that promote biliary epithelial proliferation and fibrosis (Sato et al., 2018). Next, TAMs secrete VEGF and other factors that induce angiogenesis (Roy et al., 2019). Besides, TAMs motivate CCA cells proliferation via Wnt/β-catenin signaling pathway (Loilome et al., 2014; Boulter et al., 2015). Last, TAMs inhibit T cells’ regular functions of clearing malignant cells and are associated with tumor progression, leading to a poor prognosis for CCA (Doedens et al., 2010).

A latest work was published in Januray 2024. Gao and colleagues conducted single cell transcriptomic sequencing on pre- and post-therapy iCCA patients of combination of gemcitabine with oxaliplatin and lenvatinib and anti-PD1 antibody (Lu et al., 2024). This work performed comparison between poor response group and efficient response group. The proliferation of CD8 and the transition of CD8 GZMB + to CD8 GZMK + improves response when going into the therapy, while Macro CD5L + could reduce the response by increasing CLTA-4 in CD8 GZMB+. This study underscores the impact of CD8^+^ T cell status transition and Macro CD5L + induced exhaustion in affecting response in combination treatment.

Tregs typically possess potent immunosuppressive properties and are frequently found in tumor-adjacent regions. These cells can secrete inflammatory cytokines and mediate immunosuppression by metabolizing ATP in the microenvironment (Ohta et al., 2006; Sawant et al., 2019; Guo et al., 2021; Schneider et al., 2021; Moreau et al., 2022). There is evidence that Tregs in CCA express CTLA-4 associated protein genes, which may contribute to their immunosuppressive properties, as CTLA-4 can inhibit the activation of CD8^+^ T cells by binding with CD80 expressed on antigen-presenting cells (Ma et al., 2019).

Several single-cell RNA (scRNA) sequencings have been performed and reported recently and these scRNA sequencings have revealed the CCA heterogeneity from single cell level. Since single-cell sequencing provides resolution at the individual cell level (Song et al., 2022), it has become an ideal method of analyzing heterogeneity than bulk sequencing. Kwong’s lab published their scRNA dataset (Carapeto et al., 2022). Their work involved spatial sequencing technique and therefore depicts the correlation between the immune profiling and genomic mutations. In addition, the roles played by different cell populations in CCA have also been investigated. A study conducted by Beijing University of Technology performed scRNA sequencing on 56,871 cells for 8 cases. This research illustrated the heterogeneity of fibroblasts through the transcriptomic profiles and intercellular interactions and identified fibroblast subgroups according to scRNA clustering analyses (Zhang et al., 2020). Zhang’s lab revealed distinct fibroblast subgroups first on the single cell level, and they brought inspirations to future research on this topic.

### Therapy resistance implications: spatial transcriptome

The integration of spatial transcriptomics technology has provided valuable spatial insights into the mechanisms of cholangiocarcinoma treatment resistance. A recent study published in Gut focused on patients with intrahepatic cholangiocarcinoma (iCCA) who had undergone chemotherapy, characterizing the transcriptomic landscapes that differentiate rapid progression (RP) from long survival (LS) groups (O'Rourke et al., 2024). The research team conducted diagnostic biopsies and combined these with whole transcriptome sequencing of macrodissected tissue regions from different geographic areas of the tumor for analysis. Tumor tissues were categorized into the tumor core, tumor stroma, invasive fronts, and non-tumor areas, allowing for the explicit capture of spatial expression differences. This approach identified two potential mechanisms undermining chemotherapy efficacy: enhanced immunogenic cell death and metabolic deactivation. The study also highlighted the role of bone marrow cell and T cell communication in forming an immunosuppressive environment within the RP group. The identification of an RPLS signature through spatial transcriptomics was validated across multiple cell lines, single-cell RNA sequencing data, animal models, and transcriptomic datasets, demonstrating that tumor-induced immunotolerance is a decisive factor in determining long-term survival post-chemotherapy.

Additionally, Lin and colleagues reported on the multi-omics analysis of different geographic regions of the tumor, presenting a dynamic classification of iCCA based on diverse levels of immune infiltration and immune escape (Lin et al., 2022). Each patient’s four to six primary tumor regions underwent comprehensive analysis through whole exome sequencing (Schneider et al.), RNA sequencing (RNA-seq), T-cell receptor sequencing (TCR-seq), and multiplex immunofluorescence assays. This analysis classified patients into sparse, mixed, and highly immune-infiltrated groups. The study found that highly infiltrated tumors exhibited high levels of immune activation and similar TCR repertoires across regions. However, T cell exhaustion and defects in antigen presentation could offset these factors. It was also noted that FGFR2 fusion was associated with a low tumor mutational burden (TMB) and low levels of immune infiltration. The significance of this work lies in its spatial dissection of iCCA patients’ immune heterogeneity, shedding light on its impact on the formation of immune escape mechanisms.

These studies have provided an accurate understanding of cholangiocarcinoma heterogeneity and the diverse roles of cell types, offering background knowledge of the driving forces behind the origin and continued progression of CCA. This may, in turn, assist in further elucidating the potential pathways through which CCA acquires drug resistance.

## Conclusion

We introduced the current status of CCA molecular target and ICI treatment and recent discovery of therapy resistance. With the identification of mutation sites and an improved understanding of the cell of origin and the pathway of tumor growth and development, the prospect of gaining better treatment effects for CCA is becoming more realistic and optimistic than ever. The combined use of targeted therapy and traditional treatment methods such chemotherapy provides great prospects for improving the therapeutic effectiveness of CCA. We also comprehensively elucidated the landscape of CCA pathology and the advances in treatment options from a perspective of single cell and spatial transcriptome techniques.

A noteworthy trend in recent years is the efficacy of combination therapy strategies in treating CCA, likely due to the limitations of targeted therapies alone. Most targeted therapies fail to extend progression-free survival (PFS) beyond 6 months, constrained by various resistance mechanisms. Future clinical trials should prioritize evaluating combination strategies that mitigate and overcome these resistance mechanisms. This approach could pave the way for significant advancements in the combined use of targeted drugs and ICI therapy. In addition, the treatment strategy of using specific target inhibitors needs to be further explored and optimized. For example, several clinical trials are evaluating the therapeutic effects of FGFR inhibitors on patients with FGFR2 fusion or rearrangement. Adopting inclusion criteria for people with specific genetic mutations would be of great value in achieving more instructive clinical results. We believe the expansion of novel studies from these perspectives may soon lift the veil on treatment resistance and increase the response rate.

## References

[B1] Abou-Alfa G. K. Macarulla T. Javle M. M. Kelley R. K. Lubner S. J. Adeva J. (2020a). Ivosidenib in IDH1-mutant, chemotherapy-refractory cholangiocarcinoma (ClarIDHy): a multicentre, randomised, double-blind, placebo-controlled, phase 3 study. Lancet Oncol. 21, 796–807. 10.1016/S1470-2045(20)30157-1 32416072 PMC7523268

[B2] Abou-Alfa G. K. Sahai V. Hollebecque A. Vaccaro G. Melisi D. Al-Rajabi R. (2020b). Pemigatinib for previously treated, locally advanced or metastatic cholangiocarcinoma: a multicentre, open-label, phase 2 study. Lancet Oncol. 21, 671–684. 10.1016/S1470-2045(20)30109-1 32203698 PMC8461541

[B3] Affo S. Nair A. Brundu F. Ravichandra A. Bhattacharjee S. Matsuda M. (2021). Promotion of cholangiocarcinoma growth by diverse cancer-associated fibroblast subpopulations. Cancer Cell 39, 883–882. 10.1016/j.ccell.2021.05.010 34129825 PMC8532387

[B4] Aoki S. Inoue K. Klein S. Halvorsen S. Chen J. Matsui A. (2022). Placental growth factor promotes tumour desmoplasia and treatment resistance in intrahepatic cholangiocarcinoma. Gut 71, 185–193. 10.1136/gutjnl-2020-322493 33431577 PMC8666816

[B5] Atkins M. B. Lee S. J. Chmielowski B. Tarhini A. A. Cohen G. I. Truong T. G. (2023). Combination dabrafenib and trametinib versus combination nivolumab and ipilimumab for patients with advanced BRAF-mutant melanoma: the DREAMseq trial-ECOG-ACRIN EA6134. J. Clin. Oncol. 41, 186–197. 10.1200/JCO.22.01763 36166727 PMC9839305

[B6] Balogh J. Victor III D. Asham E. H. Burroughs S. G. Boktour M. Saharia A. (2016). Hepatocellular carcinoma: a review. J. Hepatocell. carcinoma 3, 41–53. 10.2147/JHC.S61146 27785449 PMC5063561

[B7] Banales J. M. Cardinale V. Macias R. Andersen J. B. Braconi C. Carpino G. (2019). Cholangiocarcinoma: state‐of‐the‐art knowledge and challenges. Liver Int. 39, 5–6. 10.1111/liv.14101 31111668

[B8] Bekaii-Saab T. S. Valle J. W. Van Cutsem E. Rimassa L. Furuse J. Ioka T. (2020). FIGHT-302: phase III study of first-line (1L) pemigatinib (PEM) versus gemcitabine (GEM) plus cisplatin (CIS) for cholangiocarcinoma (CCA) with FGFR2 fusions or rearrangements. American Society of Clinical Oncology. 10.2217/fon-2020-0429 PMC9892961 32677452

[B9] Bertuccio P. Malvezzi M. Carioli G. Hashim D. Boffetta P. El-Serag H. B. (2019). Global trends in mortality from intrahepatic and extrahepatic cholangiocarcinoma. J. hepatology 71, 104–114. 10.1016/j.jhep.2019.03.013 30910538

[B10] Boscoe A. N. Rolland C. Kelley R. K. (2019). Frequency and prognostic significance of isocitrate dehydrogenase 1 mutations in cholangiocarcinoma: a systematic literature review. J. Gastrointest. Oncol. 10, 751–765. 10.21037/jgo.2019.03.10 31392056 PMC6657309

[B11] Boulter L. Guest R. V. Kendall T. J. Wilson D. H. Wojtacha D. Robson A. J. (2015). WNT signaling drives cholangiocarcinoma growth and can be pharmacologically inhibited. J. Clin. investigation 125, 1269–1285. 10.1172/JCI76452 PMC4362247 25689248

[B12] Bridgewater J. Galle P. R. Khan S. A. Llovet J. M. Park J.-W. Patel T. (2014). Guidelines for the diagnosis and management of intrahepatic cholangiocarcinoma. J. hepatology 60, 1268–1289. 10.1016/j.jhep.2014.01.021 24681130

[B13] Brindley P. J. Bachini M. Ilyas S. I. Khan S. A. Loukas A. Sirica A. E. (2021). Cholangiocarcinoma. Nat. Rev. Dis. Prim. 7, 65. 10.1038/s41572-021-00300-2 34504109 PMC9246479

[B14] Byron S. A. Chen H. Wortmann A. Loch D. Gartside M. G. Dehkhoda F. (2013). The N550K/H mutations in FGFR2 confer differential resistance to PD173074, dovitinib, and ponatinib ATP-competitive inhibitors. Neoplasia 15, 975–988. 10.1593/neo.121106 23908597 PMC3730048

[B15] Cadamuro M. Brivio S. Mertens J. Vismara M. Moncsek A. Milani C. (2019). Platelet-derived growth factor-D enables liver myofibroblasts to promote tumor lymphangiogenesis in cholangiocarcinoma. J. Hepatol. 70, 700–709. 10.1016/j.jhep.2018.12.004 30553841 PMC10878126

[B16] Carapeto F. Bozorgui B. Shroff R. T. Chagani S. Solis Soto L. Foo W. C. (2022). The immunogenomic landscape of resected intrahepatic cholangiocarcinoma. Hepatology 75, 297–308. 10.1002/hep.32150 34510503 PMC8766948

[B17] Chen X. Calvisi D. F. (2014). Hydrodynamic transfection for generation of novel mouse models for liver cancer research. Am. J. Pathol. 184, 912–923. 10.1016/j.ajpath.2013.12.002 24480331 PMC3969989

[B18] Cleary J. M. Iyer G. Oh D.-Y. Mellinghoff I. K. Goyal L. Ng M. C. (2020). Final results from the phase I study expansion cohort of the selective FGFR inhibitor Debio 1,347 in patients with solid tumors harboring an FGFR gene fusion. American Society of Clinical Oncology.

[B19] Cleary J. M. Raghavan S. Wu Q. Li Y. Y. Spurr L. F. Gupta H. V. (2021). FGFR2 extracellular domain in-frame deletions are therapeutically targetable genomic alterations that function as oncogenic drivers in cholangiocarcinoma. Cancer Discov. 11, 2488–2505. 10.1158/2159-8290.CD-20-1669 33926920 PMC8690974

[B20] Cortés M. Sanchez-Moral L. De Barrios O. Fernández-Aceñero M. J. Martínez-Campanario M. C. Esteve-Codina A. (2017). Tumor-associated macrophages (TAMs) depend on ZEB1 for their cancer-promoting roles. Embo J. 36, 3336–3355. 10.15252/embj.201797345 29038174 PMC5686549

[B21] Doedens A. L. Stockmann C. Rubinstein M. P. Liao D. Zhang N. Denardo D. G. (2010). Macrophage expression of hypoxia-inducible factor-1 alpha suppresses T-cell function and promotes tumor progression. Cancer Res. 70, 7465–7475. 10.1158/0008-5472.CAN-10-1439 20841473 PMC2948598

[B22] Fabris L. Perugorria M. J. Mertens J. Björkström N. K. Cramer T. Lleo A. (2019). The tumour microenvironment and immune milieu of cholangiocarcinoma. Liver Int. 39, 63–78. 10.1111/liv.14098 30907492 PMC10878127

[B23] Fabris L. Sato K. Alpini G. Strazzabosco M. (2021). The tumor microenvironment in cholangiocarcinoma progression. Hepatology 73, 75–85. 10.1002/hep.31410 32500550 PMC7714713

[B24] Farshidfar F. Zheng S. Gingras M. C. Newton Y. Shih J. Robertson A. G. (2017). Integrative genomic analysis of cholangiocarcinoma identifies distinct IDH-mutant molecular profiles. Cell Rep. 18, 2780–2794. 10.1016/j.celrep.2017.02.033 28297679 PMC5493145

[B25] Fischer C. Jonckx B. Mazzone M. Zacchigna S. Loges S. Pattarini L. (2007). Anti-PlGF inhibits growth of VEGF (R)-inhibitor-resistant tumors without affecting healthy vessels. Cell 131, 463–475. 10.1016/j.cell.2007.08.038 17981115

[B26] Franklin R. A. Li M. O. (2016). Ontogeny of tumor-associated macrophages and its implication in cancer regulation. Trends cancer 2, 20–34. 10.1016/j.trecan.2015.11.004 26949745 PMC4772875

[B27] Gao Q. Zhao Y. J. Wang X. Y. Guo W. J. Gao S. Wei L. (2014). Activating mutations in PTPN3 promote cholangiocarcinoma cell proliferation and migration and are associated with tumor recurrence in patients. Gastroenterology 146, 1397–1407. 10.1053/j.gastro.2014.01.062 24503127

[B28] Ghedini G. C. Ronca R. Presta M. Giacomini A. (2018). Future applications of FGF/FGFR inhibitors in cancer. Expert Rev. anticancer Ther. 18, 861–872. 10.1080/14737140.2018.1491795 29936878

[B29] Gingold J. A. Zhu D. Lee D.-F. Kaseb A. Chen J. (2018). Genomic profiling and metabolic homeostasis in primary liver cancers. Trends Mol. Med. 24, 395–411. 10.1016/j.molmed.2018.02.006 29530485

[B30] Golino J. L. Wang X. Maeng H. M. Xie C. (2023). Revealing the heterogeneity of the tumor ecosystem of cholangiocarcinoma through single-cell transcriptomics. Cells 12, 862. 10.3390/cells12060862 36980203 PMC10047686

[B31] Goyal L. Kongpetch S. Crolley V. E. Bridgewater J. (2021a). Targeting FGFR inhibition in cholangiocarcinoma. Cancer Treat. Rev. 95, 102170. 10.1016/j.ctrv.2021.102170 33735689

[B32] Goyal L. Meric-Bernstam F. Hollebecque A. Morizane C. Valle J. W. Karasic T. B. (2021b). Abstract CT010: primary results of phase 2 FOENIX-CCA2: the irreversible FGFR1-4 inhibitor futibatinib in intrahepatic cholangiocarcinoma (iCCA) with FGFR2 fusions/rearrangements. Cancer Res. 81, CT010. 10.1158/1538-7445.am2021-ct010

[B33] Goyal L. Meric-Bernstam F. Hollebecque A. Valle J. W. Morizane C. Karasic T. B. (2020). FOENIX-CCA2: a phase II, open-label, multicenter study of futibatinib in patients (pts) with intrahepatic cholangiocarcinoma (iCCA) harboring FGFR2 gene fusions or other rearrangements. American Society of Clinical Oncology.

[B34] Goyal L. Meric-Bernstam F. Hollebecque A. Valle J. W. Morizane C. Karasic T. B. (2023). Futibatinib for FGFR2-rearranged intrahepatic cholangiocarcinoma. N. Engl. J. Med. 388, 228–239. 10.1056/NEJMoa2206834 36652354

[B35] Goyal L. Saha S. K. Liu L. Y. Siravegna G. Leshchiner I. Ahronian L. G. (2017). Polyclonal secondary FGFR2 mutations drive acquired resistance to FGFR inhibition in patients with FGFR2 fusion–positive cholangiocarcinoma. Cancer Discov. 7, 252–263. 10.1158/2159-8290.CD-16-1000 28034880 PMC5433349

[B36] Goyal L. Shi L. Liu L. Y. Fece De La Cruz F. Lennerz J. K. Raghavan S. (2019). TAS-120 overcomes resistance to ATP-competitive FGFR inhibitors in patients with FGFR2 fusion–positive intrahepatic cholangiocarcinoma. Cancer Discov. 9, 1064–1079. 10.1158/2159-8290.CD-19-0182 31109923 PMC6677584

[B37] Green C. J. Lichtlen P. Huynh N. T. Yanovsky M. Laderoute K. R. Schaffner W. (2001). Placenta growth factor gene expression is induced by hypoxia in fibroblasts: a central role for metal transcription factor-1. Cancer Res. 61, 2696–2703. 11289150

[B38] Guo Y. Xie Y.-Q. Gao M. Zhao Y. Franco F. Wenes M. (2021). Metabolic reprogramming of terminally exhausted CD8+ T cells by IL-10 enhances anti-tumor immunity. Nat. Immunol. 22, 746–756. 10.1038/s41590-021-00940-2 34031618 PMC7610876

[B39] Hadfield M. J. Decarli K. Bash K. Sun G. Almhanna K. (2023). Current and emerging therapeutic targets for the treatment of cholangiocarcinoma: an updated review. Int. J. Mol. Sci. 25, 543. 10.3390/ijms25010543 38203714 PMC10779232

[B40] Harding J. J. Khalil D. N. Fabris L. Abou-Alfa G. K. (2023). Rational development of combination therapies for biliary tract cancers. J. Hepatology 78, 217–228. 10.1016/j.jhep.2022.09.004 PMC11111174 36150578

[B41] Heindryckx F. Coulon S. Terrie E. Casteleyn C. Stassen J.-M. Geerts A. (2013). The placental growth factor as a target against hepatocellular carcinoma in a diethylnitrosamine-induced mouse model. J. hepatology 58, 319–328. 10.1016/j.jhep.2012.09.032 23046674

[B42] Jain R. K. Xu L. (2007). alphaPlGF: a new kid on the antiangiogenesis block. Cell 131, 443–445. 10.1016/j.cell.2007.10.023 17981110

[B43] Javle M. M. Roychowdhury S. Kelley R. K. Sadeghi S. Macarulla T. Waldschmidt D. T. (2021b). Final results from a phase II study of infigratinib (BGJ398), an FGFR-selective tyrosine kinase inhibitor, in patients with previously treated advanced cholangiocarcinoma harboring an FGFR2 gene fusion or rearrangement. American Society of Clinical Oncology.

[B44] Javle M. Lowery M. Shroff R. T. Weiss K. H. Springfeld C. Borad M. J. (2018). Phase II study of BGJ398 in patients with FGFR-altered advanced cholangiocarcinoma. J. Clin. Oncol. 36, 276–282. 10.1200/JCO.2017.75.5009 29182496 PMC6075847

[B45] Javle M. Roychowdhury S. Kelley R. K. Sadeghi S. Macarulla T. Weiss K. H. (2021a). Infigratinib (BGJ398) in previously treated patients with advanced or metastatic cholangiocarcinoma with FGFR2 fusions or rearrangements: mature results from a multicentre, open-label, single-arm, phase 2 study. Lancet Gastroenterology Hepatology 6, 803–815. 10.1016/S2468-1253(21)00196-5 34358484

[B46] Kelley R. K. Bridgewater J. Gores G. J. Zhu A. X. (2020). Systemic therapies for intrahepatic cholangiocarcinoma. J. Hepatol. 72, 353–363. 10.1016/j.jhep.2019.10.009 31954497

[B47] Khan K. A. Kerbel R. S. (2018). Improving immunotherapy outcomes with anti-angiogenic treatments and vice versa. Nat. Rev. Clin. Oncol. 15, 310–324. 10.1038/nrclinonc.2018.9 29434333

[B48] Krook M. A. Bonneville R. Chen H.-Z. Reeser J. W. Wing M. R. Martin D. M. (2019). Tumor heterogeneity and acquired drug resistance in FGFR2-fusion-positive cholangiocarcinoma through rapid research autopsy. Mol. Case Stud. 5, a004002. 10.1101/mcs.a004002 PMC6672025 31371345

[B49] Lamarca A. Barriuso J. Mcnamara M. G. Valle J. W. (2020a). Molecular targeted therapies: ready for “prime time” in biliary tract cancer. J. hepatology 73, 170–185. 10.1016/j.jhep.2020.03.007 32171892

[B50] Lamarca A. Edeline J. Goyal L. (2022). How I treat biliary tract cancer. ESMO open 7, 100378. 10.1016/j.esmoop.2021.100378 35032765 PMC8762076

[B51] Lamarca A. Edeline J. Mcnamara M. G. Hubner R. A. Nagino M. Bridgewater J. (2020b). Current standards and future perspectives in adjuvant treatment for biliary tract cancers. Cancer Treat. Rev. 84, 101936. 10.1016/j.ctrv.2019.101936 31986437

[B52] Lin Y. Peng L. Dong L. Liu D. Ma J. Lin J. (2022). Geospatial immune heterogeneity reflects the diverse tumor-immune interactions in intrahepatic cholangiocarcinoma. Cancer Discov. 12, 2350–2371. 10.1158/2159-8290.CD-21-1640 35853232

[B53] Liu P. C. Koblish H. Wu L. Bowman K. Diamond S. Dimatteo D. (2020). INCB054828 (pemigatinib), a potent and selective inhibitor of fibroblast growth factor receptors 1, 2, and 3, displays activity against genetically defined tumor models. PLoS One 15, e0231877. 10.1371/journal.pone.0231877 32315352 PMC7313537

[B54] Loilome W. Bungkanjana P. Techasen A. Namwat N. Yongvanit P. Puapairoj A. (2014). Activated macrophages promote Wnt/β-catenin signaling in cholangiocarcinoma cells. Tumor Biol. 35, 5357–5367. 10.1007/s13277-014-1698-2 PMC4862210 24549785

[B55] Lu J. C. Wu L. L. Sun Y. N. Huang X. Y. Gao C. Guo X. J. (2024). Macro CD5L(+) deteriorates CD8(+)T cells exhaustion and impairs combination of Gemcitabine-Oxaliplatin-Lenvatinib-anti-PD1 therapy in intrahepatic cholangiocarcinoma. Nat. Commun. 15, 621. 10.1038/s41467-024-44795-1 38245530 PMC10799889

[B56] Ma L. Hernandez M. O. Zhao Y. Mehta M. Tran B. Kelly M. (2019). Tumor cell biodiversity drives microenvironmental reprogramming in liver cancer. Cancer Cell 36, 418–430. 10.1016/j.ccell.2019.08.007 31588021 PMC6801104

[B57] Ma L. Wang L. Chang C.-W. Heinrich S. Dominguez D. Forgues M. (2020). Single-cell atlas of tumor clonal evolution in liver cancer. bioRxiv. 10.1016/j.jhep.2021.06.028

[B58] Marabelle A. Le D. T. Ascierto P. A. Di Giacomo A. M. De Jesus-Acosta A. Delord J.-P. (2020). Efficacy of pembrolizumab in patients with noncolorectal high microsatellite instability/mismatch repair–deficient cancer: results from the phase II KEYNOTE-158 study. J. Clin. Oncol. 38, 1–10. 10.1200/JCO.19.02105 31682550 PMC8184060

[B59] Moehler M. Maderer A. Schimanski C. Kanzler S. Denzer U. Kolligs F. (2014). Gemcitabine plus sorafenib versus gemcitabine alone in advanced biliary tract cancer: a double-blind placebo-controlled multicentre phase II AIO study with biomarker and serum programme. Eur. J. Cancer 50, 3125–3135. 10.1016/j.ejca.2014.09.013 25446376

[B60] Moeini A. Sia D. Zhang Z. Camprecios G. Stueck A. Dong H. (2017). Mixed hepatocellular cholangiocarcinoma tumors: cholangiolocellular carcinoma is a distinct molecular entity. J. Hepatol. 66, 952–961. 10.1016/j.jhep.2017.01.010 28126467

[B61] Montal R. Sia D. Montironi C. Leow W. Q. Esteban-Fabró R. Pinyol R. (2020). Molecular classification and therapeutic targets in extrahepatic cholangiocarcinoma. J. Hepatol. 73, 315–327. 10.1016/j.jhep.2020.03.008 32173382 PMC8418904

[B62] Moreau J. M. Velegraki M. Bolyard C. Rosenblum M. D. Li Z. (2022). Transforming growth factor–β1 in regulatory T cell biology. Sci. Immunol. 7, eabi4613. 10.1126/sciimmunol.abi4613 35302863 PMC10552796

[B63] Norsworthy K. J. Luo L. Hsu V. Gudi R. Dorff S. E. Przepiorka D. (2019). FDA approval summary: ivosidenib for relapsed or refractory acute myeloid leukemia with an isocitrate dehydrogenase-1 mutation. Clin. Cancer Res. 25, 3205–3209. 10.1158/1078-0432.CCR-18-3749 30692099

[B64] Oh D.-Y. He A. R. Qin S. Chen L.-T. Okusaka T. Vogel A. (2022). A phase 3 randomized, double-blind, placebo-controlled study of durvalumab in combination with gemcitabine plus cisplatin (GemCis) in patients (pts) with advanced biliary tract cancer (BTC): topaz-1. American Society of Clinical Oncology.

[B65] Ohta A. Gorelik E. Prasad S. J. Ronchese F. Lukashev D. Wong M. K. (2006). A2A adenosine receptor protects tumors from antitumor T cells. Proc. Natl. Acad. Sci. 103, 13132–13137. 10.1073/pnas.0605251103 16916931 PMC1559765

[B66] O'Rourke C. J. Salati M. Rae C. Carpino G. Leslie H. Pea A. (2024). Molecular portraits of patients with intrahepatic cholangiocarcinoma who diverge as rapid progressors or long survivors on chemotherapy. Gut 73, 496–508. 10.1136/gutjnl-2023-330748 37758326 PMC10894814

[B67] Palmer W. C. Patel T. (2012). Are common factors involved in the pathogenesis of primary liver cancers? A meta-analysis of risk factors for intrahepatic cholangiocarcinoma. J. hepatology 57, 69–76. 10.1016/j.jhep.2012.02.022 PMC3804834 22420979

[B68] Park J. O. Feng Y.-H. Chen Y.-Y. Su W.-C. Oh D.-Y. Shen L. (2019). Updated results of a phase IIa study to evaluate the clinical efficacy and safety of erdafitinib in Asian advanced cholangiocarcinoma (CCA) patients with FGFR alterations. American Society of Clinical Oncology.

[B69] Perez-Fidalgo J. Martinelli E. (2023). Lenvatinib plus pembrolizumab a new effective combination of targeted agents. ESMO open 8, 101157. 10.1016/j.esmoop.2023.101157 36863093 PMC10011194

[B70] Primrose J. N. Fox R. P. Palmer D. H. Malik H. Z. Prasad R. Mirza D. (2019). Capecitabine compared with observation in resected biliary tract cancer (BILCAP): a randomised, controlled, multicentre, phase 3 study. Lancet Oncol. 20, 663–673. 10.1016/S1470-2045(18)30915-X 30922733

[B71] Razumilava N. Gores G. J. (2014). Cholangiocarcinoma. Lancet 383, 2168–2179. 10.1016/S0140-6736(13)61903-0 24581682 PMC4069226

[B72] Rolny C. Mazzone M. Tugues S. Laoui D. Johansson I. Coulon C. (2011). HRG inhibits tumor growth and metastasis by inducing macrophage polarization and vessel normalization through downregulation of PlGF. Cancer Cell 19, 31–44. 10.1016/j.ccr.2010.11.009 21215706

[B73] Roy S. Glaser S. Chakraborty S. (2019). Inflammation and progression of cholangiocarcinoma: role of angiogenic and lymphangiogenic mechanisms. Front. Med. 6, 293. 10.3389/fmed.2019.00293 PMC6930194 31921870

[B74] Sato K. Meng F. Giang T. Glaser S. Alpini G. (2018). Mechanisms of cholangiocyte responses to injury. Biochimica Biophysica Acta (BBA)-Molecular Basis Dis. 1864, 1262–1269. 10.1016/j.bbadis.2017.06.017 PMC5742086 28648950

[B75] Sato K. Zhang W. Safarikia S. Isidan A. Chen A. M. Li P. (2021). Organoids and spheroids as models for studying cholestatic liver injury and cholangiocarcinoma. Hepatology 74, 491–502. 10.1002/hep.31653 33222247 PMC8529583

[B76] Sawant D. V. Yano H. Chikina M. Zhang Q. Liao M. Liu C. (2019). Adaptive plasticity of IL-10+ and IL-35+ Treg cells cooperatively promotes tumor T cell exhaustion. Nat. Immunol. 20, 724–735. 10.1038/s41590-019-0346-9 30936494 PMC6531353

[B77] Schneider E. Winzer R. Rissiek A. Ricklefs I. Meyer-Schwesinger C. Ricklefs F. L. (2021). CD73-mediated adenosine production by CD8 T cell-derived extracellular vesicles constitutes an intrinsic mechanism of immune suppression. Nat. Commun. 12, 5911. 10.1038/s41467-021-26134-w 34625545 PMC8501027

[B78] Semenza G. L. (2012). Hypoxia-inducible factors in physiology and medicine. Cell 148, 399–408. 10.1016/j.cell.2012.01.021 22304911 PMC3437543

[B79] Sia D. Hoshida Y. Villanueva A. Roayaie S. Ferrer J. Tabak B. (2013). Integrative molecular analysis of intrahepatic cholangiocarcinoma reveals 2 classes that have different outcomes. Gastroenterology 144, 829–840. 10.1053/j.gastro.2013.01.001 23295441 PMC3624083

[B80] Silverman I. M. Hollebecque A. Friboulet L. Owens S. Newton R. C. Zhen H. (2021). Clinicogenomic analysis of FGFR2-rearranged cholangiocarcinoma identifies correlates of response and mechanisms of resistance to pemigatinib. Cancer Discov. 11, 326–339. 10.1158/2159-8290.CD-20-0766 33218975

[B81] Simons M. Gordon E. Claesson-Welsh L. (2016). Mechanisms and regulation of endothelial VEGF receptor signalling. Nat. Rev. Mol. Cell Biol. 17, 611–625. 10.1038/nrm.2016.87 27461391

[B82] Song G. Shi Y. Meng L. Ma J. Huang S. Zhang J. (2022). Single-cell transcriptomic analysis suggests two molecularly subtypes of intrahepatic cholangiocarcinoma. Nat. Commun. 13, 1642. 10.1038/s41467-022-29164-0 35347134 PMC8960779

[B83] Subbiah V. Sahai V. Maglic D. Bruderek K. Touré B. B. Zhao S. (2023). RLY-4008, the first highly selective FGFR2 inhibitor with activity across FGFR2 alterations and resistance mutations. Cancer Discov. 13, 2012–2031. 10.1158/2159-8290.CD-23-0475 37270847 PMC10481131

[B84] Turcan S. Makarov V. Taranda J. Wang Y. Fabius A. W. Wu W. (2018). Mutant-IDH1-dependent chromatin state reprogramming, reversibility, and persistence. Nat. Genet. 50, 62–72. 10.1038/s41588-017-0001-z 29180699 PMC5769471

[B85] Turner N. Grose R. (2010). Fibroblast growth factor signalling: from development to cancer. Nat. Rev. Cancer 10, 116–129. 10.1038/nrc2780 20094046

[B86] US Food and Drug Administration (2018). FDA approves an oncology drug that targets a key genetic driver of cancer, rather than a specific type of tumor. Silver Spring, MD: Case Medical Research.

[B87] US Food and Drug Administration (2019). FDA approves entrectinib for NTRK solid tumors and ROS-1 NSCLC. Silver Spring, MD: FDA.

[B88] US Food and Drug Administration (2020). FDA approves pembrolizumab for adults and children with TMB-H solid tumors. Silver Spring, MD: US Food and Drug Administration.

[B89] US Food and Drug Administration (2021). FDA grants accelerated approval to dostarlimab-gxly for dMMR advanced solid tumors. Silver Spring, MD: US Food and Drug Administration.

[B90] US Food and Drug Administration (2022). FDA grants accelerated approval to dabrafenib in combination with trametinib for unre-sectable or metastatic solid tumors with BRAF V600E mutation. Silver Spring, MD, USA: US Food and Drug Administration.

[B91] Valle J. W. Lamarca A. Goyal L. Barriuso J. Zhu A. X. (2017). New horizons for precision medicine in biliary tract cancers. Cancer Discov. 7, 943–962. 10.1158/2159-8290.CD-17-0245 28818953 PMC5586506

[B92] Varghese A. M. Patel J. Janjigian Y. Y. Meng F. Selcuklu S. D. Iyer G. (2021). Noninvasive detection of polyclonal acquired resistance to FGFR inhibition in patients with cholangiocarcinoma harboring FGFR2 alterations. JCO Precis. Oncol. 5, 44–50. 10.1200/PO.20.00178 PMC8232836 34250419

[B93] Wang Z. Ren Z. Li R. Ge J. Zhang G. Xin Y. (2021). Multi-omics integrative bioinformatics analyses reveal long non-coding RNA modulates genomic integrity via competing endogenous RNA mechanism and serves as novel biomarkers for overall survival in lung adenocarcinoma. Front. Cell Dev. Biol. 9, 691540. 10.3389/fcell.2021.691540 34368141 PMC8339593

[B94] Wiener C. M. Booth G. Semenza G. L. (1996). In vivoexpression of mRNAs encoding hypoxia-inducible factor 1. Biochem. biophysical Res. Commun. 225, 485–488. 10.1006/bbrc.1996.1199 8753788

[B95] Williams A. S. Huang W.-Y. (2013). The analysis of microsatellite instability in extracolonic gastrointestinal malignancy. Pathology 45, 540–552. 10.1097/PAT.0b013e3283653307 24018804

[B96] Wu M. J. Shi L. Dubrot J. Merritt J. Vijay V. Wei T. Y. (2022a). Mutant IDH inhibits ifnγ-TET2 signaling to promote immunoevasion and tumor maintenance in cholangiocarcinoma. Cancer Discov. 12, 812–835. 10.1158/2159-8290.CD-21-1077 34848557 PMC8904298

[B97] Wu Q. Zhen Y. Shi L. Vu P. Greninger P. Adil R. (2022b). EGFR inhibition potentiates FGFR inhibitor therapy and overcomes resistance in FGFR2 fusion-positive cholangiocarcinoma. Cancer Discov. 12, 1378–1395. 10.1158/2159-8290.CD-21-1168 35420673 PMC9064956

[B98] Xie Y. Su N. Yang J. Tan Q. Huang S. Jin M. (2020). FGF/FGFR signaling in health and disease. Signal Transduct. Target. Ther. 5, 181. 10.1038/s41392-020-00222-7 32879300 PMC7468161

[B99] Xue R. Chen L. Zhang C. Fujita M. Li R. Yan S. M. (2019). Genomic and transcriptomic profiling of combined hepatocellular and intrahepatic cholangiocarcinoma reveals distinct molecular subtypes. Cancer Cell 35, 932–947. 10.1016/j.ccell.2019.04.007 31130341 PMC8317046

[B100] Zhang M. Yang H. Wan L. Wang Z. Wang H. Ge C. (2020). Single-cell transcriptomic architecture and intercellular crosstalk of human intrahepatic cholangiocarcinoma. J. hepatology 73, 1118–1130. 10.1016/j.jhep.2020.05.039 32505533

[B101] Zhu A. X. Macarulla T. Javle M. M. Kelley R. K. Lubner S. J. Adeva J. (2021). Final overall survival efficacy results of ivosidenib for patients with advanced cholangiocarcinoma with IDH1 mutation: the phase 3 randomized clinical ClarIDHy trial. JAMA Oncol. 7, 1669–1677. 10.1001/jamaoncol.2021.3836 34554208 PMC8461552

[B102] Zhu H. Jiang X. Zhou X. Dong X. Xie K. Yang C. (2018). Neuropilin‐1 regulated by miR‐320 contributes to the growth and metastasis of cholangiocarcinoma cells. Liver Int. 38, 125–135. 10.1111/liv.13495 28618167

[B103] Zhu Y. Kwong L. N. (2020). Insights into the origin of intrahepatic cholangiocarcinoma from mouse models. Hepatology 72, 305–314. 10.1002/hep.31200 32096245

[B104] Zhu Y. Kwong L. N. (2022). IDH1 inhibition reawakens the immune response against cholangiocarcinoma. Cancer Discov. 12, 604–605. 10.1158/2159-8290.CD-21-1643 35257150

